# A missense mutation accelerating the gating of the lysosomal Cl^−^/H^+^-exchanger ClC-7/Ostm1 causes osteopetrosis with gingival hamartomas in cattle

**DOI:** 10.1242/dmm.012500

**Published:** 2013-10-23

**Authors:** Arnaud Sartelet, Tobias Stauber, Wouter Coppieters, Carmen F. Ludwig, Corinne Fasquelle, Tom Druet, Zhiyan Zhang, Naima Ahariz, Nadine Cambisano, Thomas J. Jentsch, Carole Charlier

**Affiliations:** 1 Unit of Animal Genomics, GIGA-R and Faculty of Veterinary Medicine, University of Liège (B34), 1 Avenue de l’Hôpital, 4000-Liège (Sart Tilman), Belgium.; 2 Leibniz-Institut für Molekulare Pharmakologie (FMP) and Max-Delbrück-Centrum für Molekulare Medizin (MDC), Robert-Rössle-Strasse 10, 13125 Berlin, Germany.

**Keywords:** *CLCN7*, Hamartomas, Osteopetrosis, Lysosomal storage, Ion homeostasis, Belgian Blue cattle

## Abstract

Chloride-proton exchange by the lysosomal anion transporter ClC-7/Ostm1 is of pivotal importance for the physiology of lysosomes and bone resorption. Mice lacking either ClC-7 or Ostm1 develop a lysosomal storage disease and mutations in either protein have been found to underlie osteopetrosis in mice and humans. Some human disease-causing *CLCN7* mutations accelerate the usually slow voltage-dependent gating of ClC-7/Ostm1. However, it has remained unclear whether the fastened kinetics is indeed causative for the disease. Here we identified and characterized a new deleterious ClC-7 mutation in Belgian Blue cattle with a severe symptomatology including perinatal lethality and in most cases gingival hamartomas. By autozygosity mapping and genome-wide sequencing we found a handful of candidate variants, including a cluster of three private SNPs causing the substitution of a conserved tyrosine in the CBS2 domain of ClC-7 by glutamine. The case for ClC-7 was strengthened by subsequent examination of affected calves that revealed severe osteopetrosis. The Y750Q mutation largely preserved the lysosomal localization and assembly of ClC-7/Ostm1, but drastically accelerated its activation by membrane depolarization. These data provide first evidence that accelerated ClC-7/Ostm1 gating per se is deleterious, highlighting a physiological importance of the slow voltage-activation of ClC-7/Ostm1 in lysosomal function and bone resorption.

## INTRODUCTION

Osteopetrosis, also known as marble bone disease, is a heterologous group of inherited disorders that is characterized by fragile bones, generally caused by an impaired bone resorption by osteoclasts (Tolar et al., 2004). Children affected by severe osteopetrosis are often blind due to compression of the optical nerve, and the patients usually die within the first decade of life as a result of secondary defects caused by bone marrow insufficiency. Two of the genes whose mutations cause osteopetrosis are *CLCN7* and *OSTM1*, encoding the chloride-proton (Cl^−^/H^+^) exchanger ClC-7 and its obligate β-subunit Ostm1 (osteopetrosis-associated transmembrane protein 1) (Chalhoub et al., 2003; Kornak et al., 2001; Lange et al., 2006; Leisle et al., 2011). At least in mice, dysfunction of ClC-7/Ostm1 additionally leads to a neuronal ceroid lipofuscinosis (NCL)-like lysosomal pathology (Kasper et al., 2005; Lange et al., 2006), and a severe form of osteopetrosis is accompanied by neurodegeneration and accumulation of ceroid lipofuscin in humans (Steward, 2003).

ClC-7 belongs to the CLC family of chloride channels and transporters, which consists of nine mammalian members with diverse physiological roles (Stauber et al., 2012). The CLC family comprises both plasma membrane-localized chloride channels and chloride–proton exchangers that reside predominantly on compartments of the endocytic pathway (Jentsch, 2008; Stauber et al., 2012). ClC-7 and its β-subunit Ostm1 localize to lysosomes of all cells and additionally reside at the ruffled border membrane of bone-resorbing osteoclasts (Kornak et al., 2001; Lange et al., 2006). The latter specialized plasma membrane domain is built up by lysosomal exocytosis and serves to acidify the resorption lacuna, a process required for the degradation of bone material (Teitelbaum, 2000). Both the formation of the ruffled border and the acidification of the resorption lacuna depend on functional ClC-7/Ostm1 (Kornak et al., 2001; Lange et al., 2006). Hence, dysfunction of ClC-7/Ostm1 leads to osteopetrosis in mice and humans (Chalhoub et al., 2003; Kornak et al., 2001; Lange et al., 2006). Contrasting with the impact on the pH of the resorption lacuna, the lysosomal pH is not changed in cells lacking either ClC-7 or Ostm1 (Kasper et al., 2005; Lange et al., 2006; Steinberg et al., 2010; Weinert et al., 2010). In spite of normal lysosomal pH, lysosomal degradation of endocytosed protein is impaired in ClC-7/Ostm1-deficient mice (Wartosch et al., 2009), which develop a neurodegenerative lysosomal storage disease in addition to osteopetrosis (Kasper et al., 2005; Lange et al., 2006; Pressey et al., 2010). Like the other intracellular CLCs, ClC-7/Ostm1 mediates voltage-dependent Cl^−^/H^+^ exchange, but is unique in its slow activation and deactivation in response to voltage steps (Leisle et al., 2011). Intriguingly, several osteopetrosis-causing human mutations accelerate this gating process (Leisle et al., 2011). Many of these accelerating mutations change amino acids close to the interface between cytosolic CBS domains and the transmembrane part of ClC-7, suggesting that a physical interaction between these parts is involved in ClC-7 gating (Leisle et al., 2011). However, as we lacked data on the expression levels and subcellular localization of the mutant proteins in affected tissues, it remains unclear whether accelerated gating per se causes the human pathology.

The Belgian Blue cattle breed (BBCB; *Bos taurus*) is a beef breed from Belgium, famous for its hyper-muscled appearance caused by a spontaneous myostatin knockout allele (Grobet et al., 1997). The BBCB population comprises approximately half a million cows and 2500 registered bulls. Extensive use of artificial insemination (about half of births), associated with intense selection for traits related to meat production, contracts the effective population size (*N*_e_ ~60), thereby causing frequent outbursts of recessive defects.

**TRANSLATIONAL IMPACT**

**Clinical issue**

Osteopetrosis is characterized by an increase in bone density owing to a failure in bone resorption (the breakdown of bone to release minerals into the bloodstream) by osteoclasts. Severe osteopetrosis is commonly accompanied by anemia and susceptibility to infections because of insufficient hematopoiesis in the obliterated medullary cavity and bone marrow narrowing. Osteopetrosis can be caused by mutations that impair the generation or the function of osteoclasts. The latter class comprises mutations in the genes *CLCN7* and *OSTM1*, which encode the lysosomal Cl^−^/H^+^ exchanger ClC-7 and its obligate β-subunit Ostm1, respectively. In addition to its ubiquitous lysosomal localization, the ClC-7/Ostm1complex is present at the ‘ruffled border’ of osteoclasts. This plasma membrane domain is built up by lysosomal exocytosis and serves to acidify the bone-facing resorption lacunae. Both the formation of the ruffled border and acid secretion across it might require ClC-7/Ostm1. Interestingly, dysfunction of lysosomal ClC-7/Ostm1 also results in a neuronal pathology that cannot be treated by bone marrow transplantation, the usual treatment for osteopetrosis. Therefore, the precise function of the ClC-7/Ostm1 complex and its relative contribution to the pathogenesis of osteopetrosis are important questions.

**Results**

In this study, the authors used whole-genome sequencing to map a new *CLCN7* mutation that underlies a recessively inherited, severe form of osteopetrosis in Belgian Blue cattle. Affected calves were mostly stillborn. X-ray imaging and sectioning revealed that long bones were hyper-mineralized and fragile as in human patients and in ClC-7/Ostm1-deficient mice; however, unlike affected humans and mice, the cattle also presented with large gingival hamartomas (benign tumor-like nodules). Surprisingly, the mutation was shown to have only a small effect on the *in vivo* expression levels and localization of ClC-7/Ostm1. Biophysical experiments revealed that the mutation did not reduce ion transport; instead it significantly accelerated the normally slow activation and deactivation of ClC-7/Ostm1-mediated Cl^−^/H^+^ exchange.

**Implications and future directions**

The authors had found previously that some human disease-causing *CLCN7* mutations accelerate the usually slow voltage-dependent activation of ClC-7/Ostm1. Because it is unknown whether these mutations also decrease ClC-7/Ostm1 protein levels in patients, it remained unknown whether the acceleration of ClC-7 is causative for osteopetrosis. The present data suggest that indeed not only loss-of-function, but also faster gating kinetics of ClC-7/Ostm1 might be deleterious, thereby revealing a new mechanism by which mutations in ClC-7/Ostm1 lead to disease of lysosomes and bones. It will be interesting to see whether the different mechanisms by which ClC-7/Ostm1 dysfunction impairs bone resorption contribute to the phenotypical variability of human osteopetrosis and why the activation of ClC-7/Ostm1 ion transport needs to be slow to support normal lysosomal function and bone resorption.

In 2005, endorsed by breeders and veterinarians, we established a heredo-surveillance platform to centralize relevant information and biological samples for emerging genetic anomalies, identify responsible genes and mutations, and develop diagnostic tests. Since then, we have mapped and identified the causative gene and mutation(s) for 12 and eight diseases, respectively, including congenital muscular dystonia I and II, crooked tail syndrome and stunted growth in BBCB (Charlier et al., 2008; Fasquelle et al., 2009; Sartelet et al., 2012a; Sartelet et al., 2012b). These successes were largely due to the development of medium-density single-nucleotide polymorphism (SNP) chips (~50 K) for cattle, allowing efficient autozygosity mapping in what equates to a small, genetically isolated population. However, like in other domestic animal species, the downside of the peculiar demography is the limited mapping resolution that can be achieved. Depending on local gene density, segments of autozygosity typically cover tens to a hundred of positional candidate genes. In the absence of obvious functional candidates, pinpointing the causative gene and mutation remains slow and laborious.

In this study, by combining medium-density SNP arrays and whole-genome sequencing (WGS) we identified a missense mutation in *CLCN7* as responsible for a symptomology in newborn BBCB calves that encompasses abnormal skull formation and often gingival hamartomas and stillbirth. Further analysis of affected calves revealed a severe osteopetrosis and signs of lysosomal storage. Although the mutation neither altered expression levels nor the localization of ClC-7, it accelerated its gating kinetics. These data strongly suggest a functional role of the slow gating kinetics of ClC-7/Ostm1 for lysosomal function and bone resorption.

## RESULTS

### The gene for gingival hamartomas maps to a 1.3 Mb interval on bovine chromosome 25

**Fig. 1.**
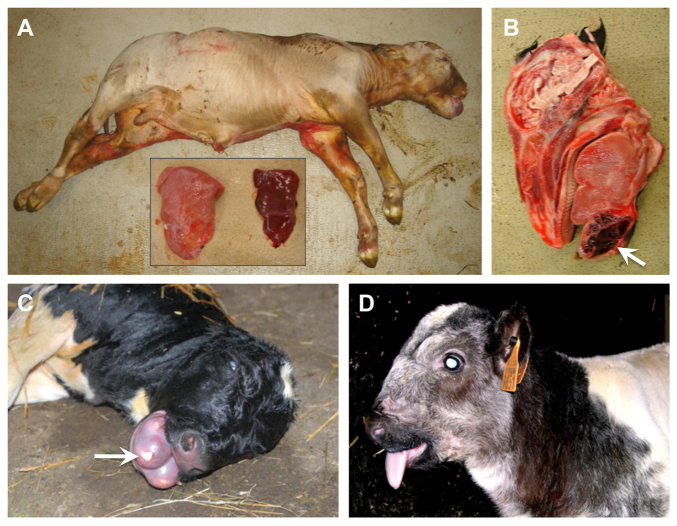
**Clinical features of congenital hamartomas in affected Belgian Blue calves.** (A) Premature stillborn calf exhibiting gingival hamartoma, abnormal skull shape, hydrops and hepatomegaly. Inset shows livers from mutant (left) and wild-type (right) calves. (B) Sagittal section of a case head revealing a hamartoma within the inferior jaw. (C) Dead case presenting a voluminous hamartoma; note teeth inclusion (arrow). (D) Alive case with abnormal skull shape accompanied by a protruding tongue.

**Fig. 2.**
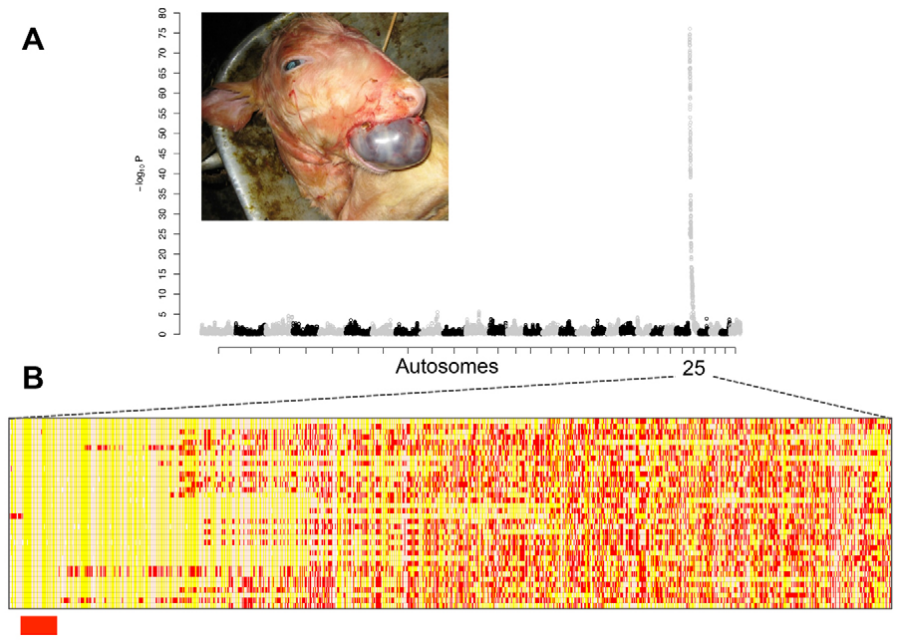
**Genome-wide association mapping for the hamartoma disease locus.** (A) Manhattan plot for case/control GWAS presenting a unique genome-wide significant signal on chromosome 25; the 29 autosomes are alternately labeled in gray or black. inset shows a typical hamartoma case. (B) Cases genotypes for 1256 chromosome 25 SNP; homozygous genotypes are shown in yellow or white, heterozygous genotypes in red; the centromeric 1.15-Mb homozygosity region, identical by state among all cases, is highlighted in red.

Between 2008 and 2010, we collected biological material with pedigree records for 63 newborn calves with shared symptomatology: affected calves were mostly stillborn (70%) or slightly premature (gestation length between 210 and 260 days; 73%) and displayed a small body size (45%) and abdominal hydrops (58%), an abnormal skull shape (100%), inferior brachygnatism (100%), protruding tongue (81%) and gingival hamartomas of variable size (up to 15 cm diameter; 80%) located on the lower jaw (Fig. 1 and supplementary material Table S1). All calves born alive were blind and consequently euthanized within days or weeks. At necropsy, we observed liver (82%; Fig. 1) and kidney (59%; not shown) hypertrophies. Mothers of affected calves typically suffered from hydramnios, a condition commonly associated with impaired swallowing in the fetus (Drost, 2007). As a result, ~50% of the dams needed to be culled after parturition, causing considerable extra losses to farmers. Examination of the pedigrees of the 63 cases suggested a recessive mode of inheritance as all of them traced back on both paternal and maternal side to a common ancestor (*Gabin d’Offoux*, a sire born in 1977). Thirty-three cases and 275 healthy controls were initially genotyped using a previously described custom bovine 50K SNP array (Charlier et al., 2008). We performed a genome-wide haplotype-based association study with a generalized linear mixed model accounting for stratification using GLASCOW (Zhang et al., 2012). We identified a single genome-wide significant signal (*P*<10^−76^) on the proximal end of chromosome 25 (BTA25) (Fig. 2A). Visual examination of the SNP genotypes defined a non-recombinant autozygous interval of 1.15 Mb [Bovine Genome assembly bTau6 (UMD3.1), chr25: 632,647-1,781,139] shared by all 33 cases and encompassing 82 annotated transcripts (Fig. 2B; supplementary material Fig. S1 and Table S2).

### Whole-genome sequencing identifies a likely causative missense mutation in *CLCN7*

**Fig. 3.**
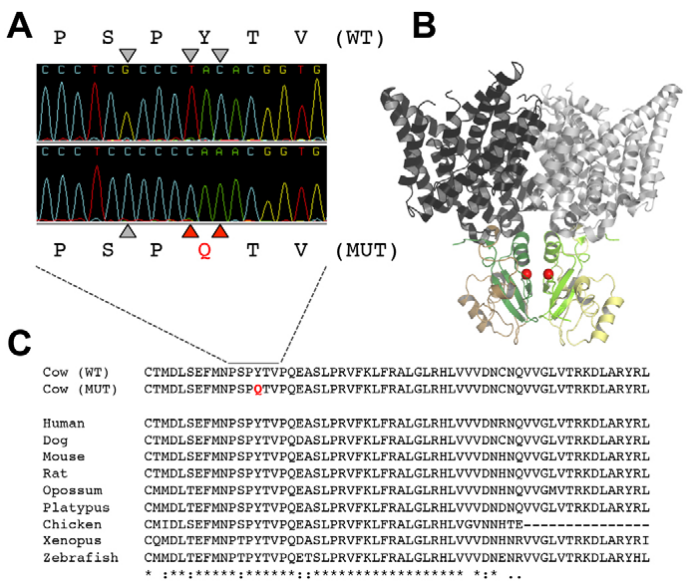
**Missense mutations in the second CBS domain of the ClC-7 protein.** (A) Sequence traces of *CLCN7* exon 23 for wild-type (top) and mutant (bottom) calves; triangles pinpoint three nucleotide substitutions with the corresponding Y750Q amino acid mutation in red. (B) X-ray structure of CmCLC displaying the localization of the mutated amino acid (red spheres in either subunit of the dimer) according to the published alignment between CmCLC and ClC-7 (Feng et al., 2010). The transmembrane core-forming parts are depicted in gray, the cytosolic domains CBS1 in yellow and CBS2 in green, using darker colors for one subunit. (C) ClC-7 CBS2 domain alignment, from mammals to fish, showing its conservation through evolution with the Y750Q mutation in red.

In the absence of any obvious candidate gene, we decided to sequence the whole genome of four affected cases, which were homozygous for the shared identical-by-descent (IBD) segment, and eight unrelated Belgian Blue controls selected as non-carriers for the disease haplotype. Individual paired-end libraries (insert size ~250 bp) were generated and ~40 genome-equivalents of paired-end reads (2×110 bp) were sequenced on an Illumina GAIIx instrument. Sequence reads were mapped to the bTau6 build using the Burrows-Wheeler Aligner (BWA) (Li and Durbin, 2009), and resulting alignments directly visualized with the Integrative Genomics Viewer (IGV) (Robinson et al., 2011). An average of ~5.4 Gb were uniquely mapped per animal, resulting in a joint ~8-and ~32-fold coverage of the region of autozygosity in cases and controls, respectively. DNA sequence variants (DSVs) were called with SAMtools (Li et al., 2009). We detected 2001 SNPs and 161 indels for a total of 2162 DSVs; 1733 of these (80%) were filtered-out because they were found in the controls or previously reported in breeds other than BBCB (W.C., personal communication). Out of the remaining 429, 111 mapped to transcribed regions, eight to ORF (Refseq annotation), of which two caused an amino acid substitution. However, none of these was considered damaging by Polyphen2 and SIFT programs for protein-sequence-based prediction of deleteriousness (Adzhubei et al., 2010; Sim et al., 2012). We further visually scrutinized the entire 1.15 Mb region of autozygosity. This revealed three sequence reads encompassing a cluster of three previously undetected nucleotide substitutions *[c2244G >C + c2248T >C + c2250C >A]* located in exon 23 of the *CLCN7* gene encoding the anion transport protein ClC-7 (supplementary material Fig. S2). Conventional Sanger sequencing of five homozygous cases validated the three DSVs (Fig. 3A). Two of these, *[c2248T >C + c2250C >A]*, jointly cause a tyrosine-to-glutamine substitution (TAC >CAA: Y750Q), whereas the third *[c2244G >C]* is silent (TCG >TCC: S748). The Y750 residue maps to the second CBS domain (CBS2) of the ClC-7 protein close to the dimer subunit interface (Fig. 3B). It is highly conserved between species (Fig. 3C) but not present in the other eight CLC paralogues (not shown). A position-specific scoring matrix (PSSM) (Marchler-Bauer et al., 2011) output for the CBS2 domain of the mutant bovine ClC-7 is presented in supplementary material Fig. S3.

Since the original analysis, we had access to 50 additional whole-genome sequences of Belgian Blue elite AI sires, of which six were acknowledged carriers of the causative mutation based on the occurrence of mutant calves in their progeny and 44 used as control because they were non-carriers of the disease haplotype. Sequence reads were mapped as described above and DSVs were called jointly for affected cases (4), carrier sires (6) and non-carrier sires (44) with the Genome Analysis Toolkit v2 (McKenna et al., 2010). Within the defined 1.15-Mb region, among a total of 4829 detected DSVs, only 16 fulfilled the two following criteria: (i) present at homozygous stage in affected cases, and (ii) present at heterozygous stage in carrier and absent in control animals. Eight were intergenic, three intronic and five coding, including the cluster of three *CLCN7* substitutions (supplementary material Table S3). The two remaining coding variants corresponded to one synonymous and one non-synonymous change. The non-synonymous variant, leading to a R1023Q in the *IFT140* gene, was observed at homozygous stage in other cattle breeds and predicted benign by Polyphen2 and SIFT. Thus, the ClC-7 Y750Q stood out as the sole putatively deleterious mutation on the disease allele.

We developed an assay to directly interrogate the *[c2248T >C + c2250C >A]* missense mutations and genotyped the 63 cases, 74 of their parents, 141 animals from 11 breeds other than BBCB, and 6489 healthy BBCB animals. All cases were homozygous for the Y750Q mutation, whereas available parents and putative founder (*Gabin*) were all carriers. The mutation was absent in the non-BBCB cohort, and detected at a frequency of 5% (644 carriers) in BBCB controls. None of the genotyped controls was homozygous for the mutant (*P*=0.000026 under Hardy-Weinberg equilibrium).

### ClC-7 causality is strengthened by severe osteopetrosis of affected calves

**Fig. 4.**
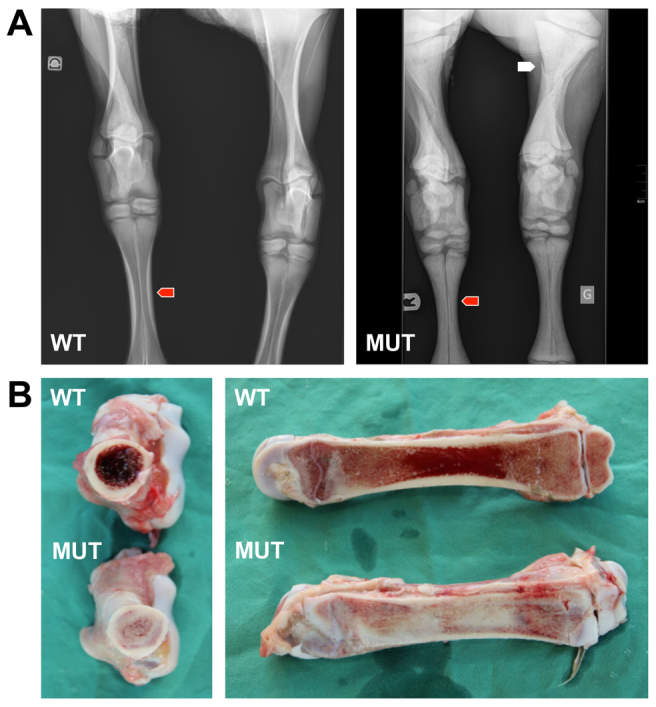
**Severe osteopetrotic phenotype exhibited by Y750Q homozygous calves.** (A) Dorsoventral radiographs of extended hind legs of a one-week-old homozygous mutant calf (MUT, right) and an age-matched Belgian Blue control calf (WT, left) are presented. X-rays were performed using a digital radiograph system [Vertix Vet (150 kV/600 mA), Siemens, Germany] with 75 kV and 100 mA as technical parameters. The bone marrow cavity is clearly visible within metatarsus of the wild-type calf but absent from the mutant corresponding bone (red arrows). The mutant calf also exhibits a tibia fracture (white arrow) probably consecutive to the acknowledged increased fragility of osteopetrotic long bones. (B) Fresh transversal (left) and sagittal (right) sections of long bones (tibia) of age-matched mutant (MUT) and control (WT) calves showing an absence of central bone marrow cavity for the mutant.

As mutations in ClC-7 can entail osteopetrosis in humans and mice [first described in Kornak et al. (Kornak et al., 2001)], we searched for related clinical symptoms in four newly referred cases shown to be homozygous for the Y750Q mutation. This revealed a previously overlooked, severe osteopetrosis of the long bones (Fig. 4). Other features typical for this condition described in human and mouse were also noticed in calves, including abnormal skull shape, small body size and visual deficiency or blindness due to retinal degeneration (listed in supplementary material Table S1). The striking gingival hamartomas displayed by the majority of mutant calves might be related to an impaired tooth eruption, which has been described for ClC-7 knockout mice (Kornak et al., 2001) and in anecdotal reports of small odontomas in human patients (Luzzi et al., 2006; Xue et al., 2012). Accordingly, the invariable location of the hamartoma on the lower jaw may be related to the absence of canine and incisor teeth on the upper jaw in cattle.

**Fig. 5.**
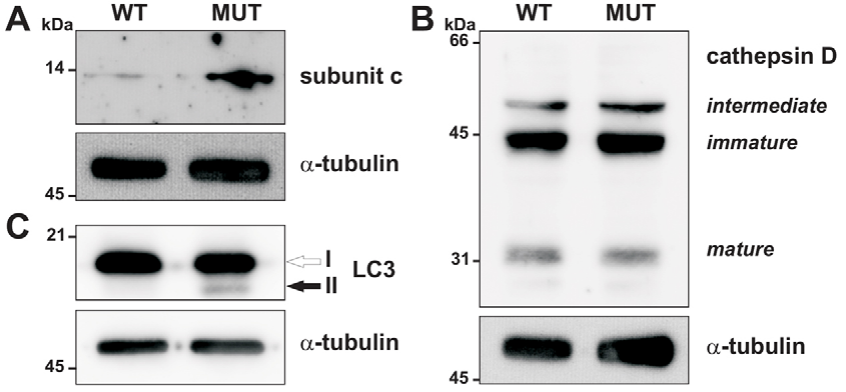
**Lysosomal storage in a homozygous ClC-7(Y750Q) calf.** Lysates (60 μg protein per lane) of cerebellum from a control calf (WT) and from a calf homozygous for the Y750Q mutation (MUT) were analyzed by immunoblot against the indicated proteins. Immunoblotting for α-tubulin served as loading control. (A) Levels of subunit c of the mitochondrial ATP synthase were increased in the mutated calf. (B) The animals displayed no differences in the levels of the preprocathepsin D (immature) or the intermediate and mature forms of cathepsin D. (C) The autophagic marker LC3-II was increased in the mutated calf whereas overall LC3 levels were unchanged.

Besides osteopetrosis, ClC-7-deficient mice develop a lysosomal storage disease and display a progressive neurodegeneration accompanied by microglial activation and astrogliosis in the central nervous system (Kasper et al., 2005; Pressey et al., 2010). To test whether we could find signs pointing to a similar pathology in cattle, we analyzed protein lysate from the available cerebellum from the only homozygous Y750Q mutant calf (4 days old) from which we had tissue samples and compared it to that of a healthy 1-month-old calf. In this unique *ex vivo* sample, we detected an increased amount of the mitochondrial ATP-synthase subunit c (Fig. 5A), which accumulates as lysosomal storage material in ClC-7-deficient mice (Kasper et al., 2005). In contrast to *Clcn7*^−/−^ mice (Kasper et al., 2005), no increase in cathepsin D levels was detected by immunoblot (Fig. 5B). Similarly to ClC-7-deficient mice (Wartosch et al., 2009), the autophagic marker LC3-II was increased in cerebellum (Fig. 5C) and kidney (not shown) of this one mutant calf compared to a control calf. Unfortunately, the available tissue was not suitable for ultrastructural or (immuno)histological analysis, so that we lack convincing evidence for a lysosomal storage disease.

Taken together, these results – mainly the osteopetrosis in affected calves – strongly support the causality of the *CLCN7* gene.

### Protein levels of ClC-7 and Ostm1 are minimally affected by the Y750Q mutation

Total RNA was extracted from kidney and cerebellum from a homozygous mutant calf and an age-matched BBCB control. The RNA was reverse transcribed and three primer pairs (specified in supplementary material Table S4) were utilized to amplify overlapping amplicons (541, 1088 and 1256 bp) covering the entire 2427 bp bovine *CLCN7* ORF. We did not observe obvious qualitative or quantitative differences between case and control amplicons upon agarose gel electrophoresis (data not shown). Sanger sequencing of the three amplicons confirmed the *[c2244G >C + c2248T >C + c2250C >A]* nucleotide substitutions as the only differences (data not shown).

**Fig. 6.**
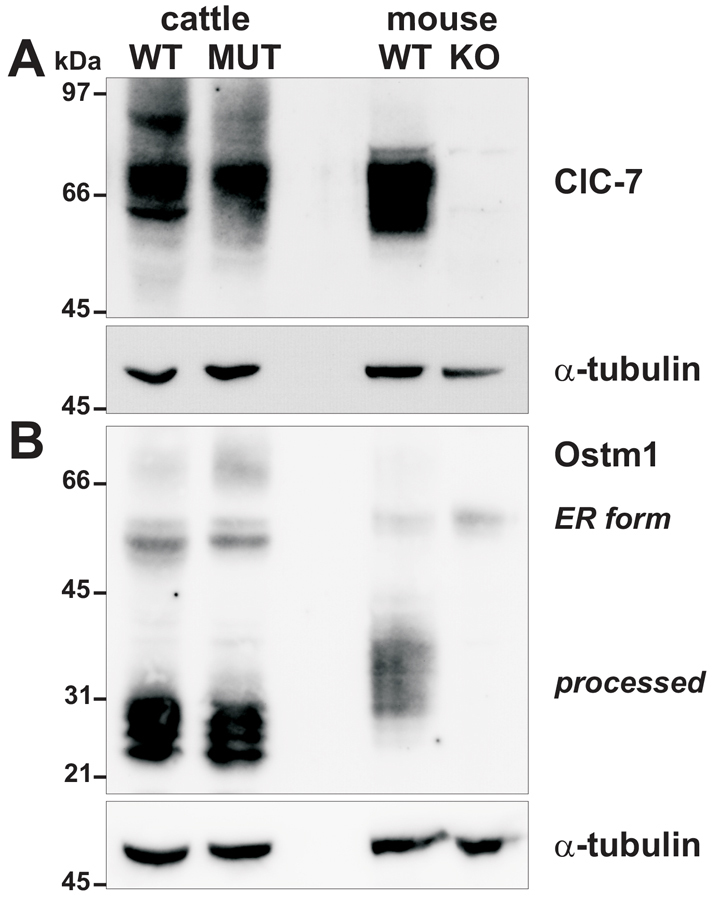
**Protein levels of ClC-7 and Ostm1.** Membrane protein-enriched lysates (80 μg per lane) of kidney from a control calf (WT) and a calf homozygous for the Y750Q mutation (MUT), as well as from a *Clcn7*^−/−^ mouse (KO) and its wild-type littermate (WT) were analyzed by western blot for ClC-7 (A) and Ostm1 (with an antibody directed against a C-terminal epitope that also recognizes the proteolytically processed transmembrane fragment) (B). Immunoblotting for α-tubulin served as loading control. Lack of ClC-7 signal in lysate from the *Clcn7*^−/−^ mouse shows the specificity of the antibody. Ostm1, which migrates with an apparently higher molecular weight in the bovine samples, exists predominantly in its proteolytically processed form (~30 kDa). The different sizes could be due to species-specific differences in glycosylation.

ClC-7 protein destabilization by a disease-causing mutation has previously been reported in a human case (Kornak et al., 2001). To investigate whether the bovine Y750Q mutation might similarly affect ClC-7 stability, we analyzed membrane protein extracts from kidney (Fig. 6A) and cerebellum (not shown) of wild-type and mutant calves by immunoblotting against ClC-7. In both types of tissue, we observed only a modest reduction in ClC-7 concentration in the mutant calf. Additionally, apart from the main ClC-7 band there are minor bands at ~60 kDa and ~85 kDa of unknown nature in the wild-type lysate that are strongly reduced in the mutant. It seems very unlikely that these differences cause the observed severe osteopetrotic phenotype of mutant calves because heterozygous *Clcn7*^+/−^ mice display no phenotype (Kornak et al., 2001). Also, for the β-subunit of ClC-7, Ostm1, only subtle differences were detected, such as the stronger staining of a smear close to ~66 kDa (Fig. 6B). We did not observe large changes in overall levels of either the endoplasmic reticulum (ER) precursor form or of the predominant processed form. By contrast, Ostm1 levels are drastically reduced in mice lacking ClC-7 (Lange et al., 2006) (Fig. 6B). To test whether changes in protein levels might be observed in heterologous expression, we inserted the corresponding mutation into an available rat ClC-7 expression vector (Y744Q in rat ClC-7) and transfected HeLa cells with Ostm1 and either wild-type or mutant ClC-7. Western blots of protein lysate from these cells revealed only subtle differences for ClC-7 and Ostm1, in stark contrast to the absence of the processed form of Ostm1 when it was overexpressed without ClC-7 (supplementary material Fig. S4).

### The Y750Q mutation does not abolish the functional interaction between ClC-7 and Ostm1 or their co-trafficking to lysosomes

**Fig. 7.**
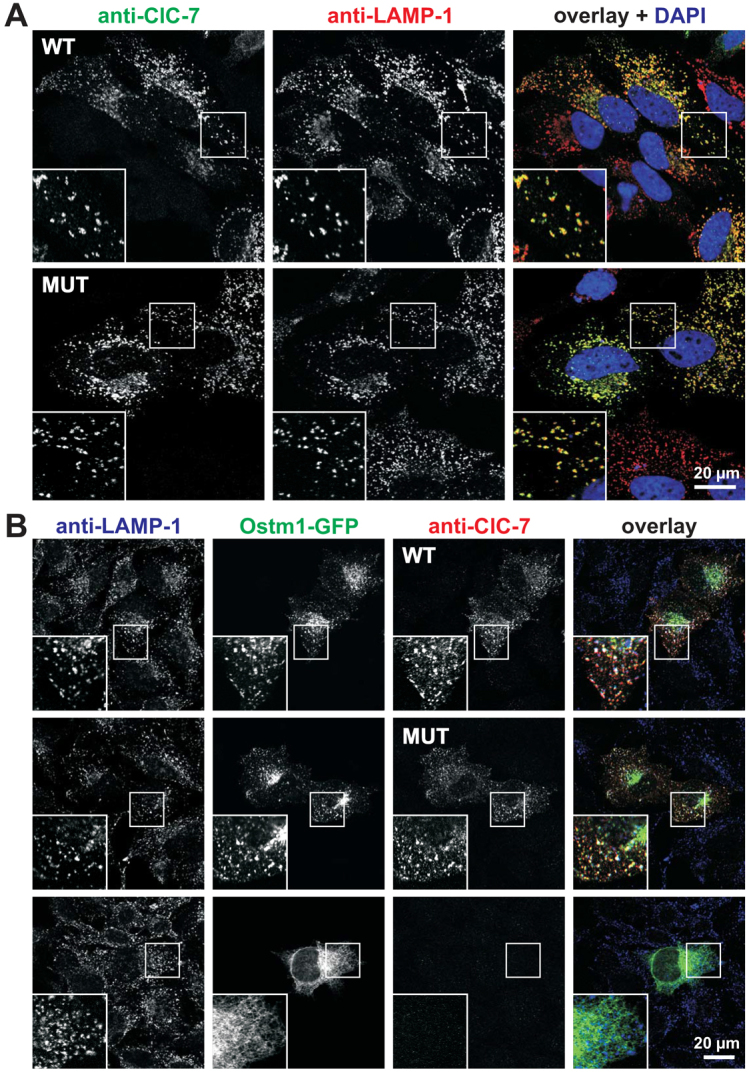
**Correct subcellular targeting of ClC-7/Ostm1 upon heterologous expression.** (A) After transient transfection with rat ClC-7, either wild-type (WT) or the Y744Q mutant (MUT), HeLa cells were immunolabeled for ClC-7 (green in overlay) and the late endosomal/lysosomal marker protein LAMP-1 (red); blue in overlay indicates DAPI staining of nuclei. (B) HeLa cells co-transfected with rat ClC-7 (either WT or MUT) and mOstm1-GFP (green in overlay), or with Ostm1-GFP alone (bottom panel) were immunolabeled for ClC-7 (red) and the late endosomal/lysosomal marker protein LAMP-1 (blue).

Some human osteopetrosis-causing *CLCN7* mutations impair trafficking of ClC-7, resulting in retention of the mutant protein in the ER (Leisle et al., 2011; Schulz et al., 2010). We evaluated potential effects of the Y750Q mutation on the subcellular targeting of heterologously expressed ClC-7. Upon transfection in HeLa cells, mutant ClC-7 was correctly targeted to lysosomes as was the wild-type ClC-7 (Fig. 7A). Ostm1, which is required for protein stability and the Cl^−^/H^+^ exchange activity of ClC-7 (Lange et al., 2006; Leisle et al., 2011), needs binding to ClC-7 for ER export and targeting to lysosomes (Lange et al., 2006; Stauber and Jentsch, 2010). To test whether the Y750Q mutation affects ClC-7/Ostm1 interaction or Ostm1 trafficking to lysosomes, wild-type and mutant ClC-7 expression vectors were co-transfected with Ostm1 that was labeled by a green-fluorescent protein (GFP) fused to its C-terminus. Immunohistochemistry of transfected cells using antibodies directed against the late endosomal/lysosomal marker protein LAMP-1 showed that Ostm1 trafficks with both wild-type and mutant ClC-7 to lysosomes, whereas Ostm1-GFP remains in the ER when expressed without ClC-7 (Fig. 7B). Furthermore, immunoblots of kidney membrane protein lysate against Ostm1 showed that the bulk of Ostm1 was proteolytically processed to its mature ~30 kDa form, with only subtle difference between wild-type and affected calves (Fig. 6B). Because cleavage occurs in or on the way to lysosomes (Lange et al., 2006), this finding provides support for correct *in vivo* targeting of Ostm1. This was confirmed by western blot analysis of overexpressed Ostm1, which can be detected at similar levels in its processed form when coexpressed with wild-type or mutant ClC-7, but not when overexpressed alone (supplementary material Fig. S4). Thus, the Y750Q mutation does not inhibit the ClC-7/Ostm1 interaction or its ClC-7-dependent trafficking to lysosomes.

### The Y750Q mutation accelerates the gating of ClC-7/Ostm1

**Fig. 8.**
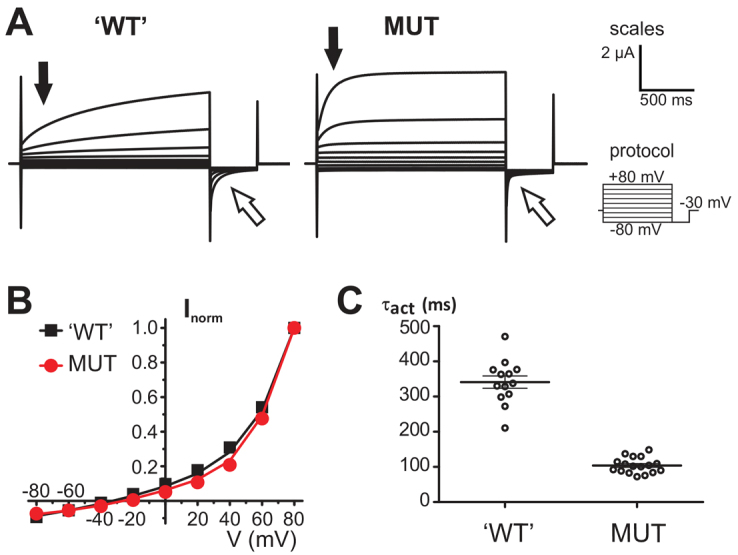
**Accelerated gating of ClC-7/Ostm1 by the disease-causing mutation.** *Xenopus* oocytes coexpressing Ostm1 and partially plasma membrane-localized hClC-7^PM^, either without further mutation (‘WT′) or carrying the Y746Q mutation (MUT), were recorded in a two-electrode voltage clamp. (A) Current traces (representative for three batches of oocytes) recorded with the clamp protocol shown on the right (holding potential −30 mV, subsequent 2-second test pulses between −80 mV and 80 mV in 20-mV intervals, each followed by a 0.5-second deactivation pulse at −80 mV) are shown as superimposition for ‘WT′ and MUT. Activation (black arrows, quantified in C) and relaxation kinetics (white arrows) of the currents were accelerated by the Y746Q mutation. Scales for the time and intensity of the current traces are shown on the right (the clamp protocol is not shown in scale). (B) Mean currents after 2 seconds were normalized to the current at +80 mV and plotted as function of voltage. Values are the mean of 13 (‘WT′) and 18 (MUT) oocytes from three batches of oocytes, small error bars (s.e.m.) are hidden behind the symbol. (C) Rate constants of current activation were determined by a single exponential fit of the current trace during the first 250 milliseconds of depolarization to 80 mV for each of the measured oocytes. Thick lines in data point clouds indicate the arithmetic mean, and thin lines the s.e.m. (*P*<10^−6^ calculated by *t*-test between ‘WT′ and MUT).

We next assessed the effect of the Y750Q mutation on ion transport by ClC-7/Ostm1. We made use of a human ClC-7 construct that is partially mislocalized to the plasma membrane (hence referred to as ClC-7^PM^) due to the disruption of two dileucine-based endosomal sorting motifs (Stauber and Jentsch, 2010). The biophysical characteristics of this surface-expressed mutant can be conveniently analyzed in *Xenopus* oocytes by two-electrode voltage-clamp recording (Leisle et al., 2011). ClC-7^PM^/Ostm1 mediates outwardly rectifying currents that are slowly gated by voltage (Leisle et al., 2011). Introduction of the mutation corresponding to Y750Q (Y746Q) did not reduce the current amplitude and had no detectable effect on the outwardly rectifying voltage dependence of ClC-7^PM^/Ostm1 (Fig. 8A,B). However, it accelerated the voltage-dependent activation and relaxation kinetics more than threefold (Fig. 8A,C), with an activation rate constant at +80 mV of 104±6 milliseconds for the Y746Q mutant versus 341±18 milliseconds for ‘wild-type’ human ClC-7^PM^/Ostm1 (mean values ± s.e.m. from 17 and 13 oocytes, respectively, from three independent batches of oocytes).

## DISCUSSION

### Identification of the disease-causing gene and mutation

Our previous identifications of genes and mutations underlying various syndromes in BBCB (Charlier et al., 2008; Fasquelle et al., 2009; Sartelet et al., 2012a; Sartelet et al., 2012b) were mainly facilitated by the use of medium-density SNP chips for bovine samples. In human genetics, molecular elucidation of monogenic disorders has benefited tremendously from next generation sequencing (NGS), either in combination with whole-exome capture or targeted capture of specific genomic regions, or used to directly sequence the entire genome (WGS) (Lupski et al., 2010; Ng et al., 2009; Volpi et al., 2010). Whole-exome sequencing was also successfully employed to identify a human *CLCN7* mutation in osteopetrosis patients (Sui et al., 2013). Until recently, targeted capture of specific genomic regions was seriously hampered in cattle as a result of the suboptimal quality of the reference genome; exome-capturing reagents were not yet available. We therefore elected to use WGS to tentatively speed up the final positional cloning step. Indeed, even if more costly, WGS was thought to generate extremely valuable ‘byproducts’, including breed-specific catalogues of common polymorphisms that could be used to filter out non-causative variants (Charlier et al., 2012). Along the same line, WGS can be mined to identify variants that are predicted to disrupt gene and/or protein function. These include splice site variants, frame-shift mutations, nonsense mutations and missense mutations predicted to be disruptive. Depending on their frequency, some of them could negatively affect the fitness of the breed. WGS also uncovered thousands of non-synonymous variants, of which a number are bound to influence traits of economic importance. These non-synonymous variants could be directly usable by breeding organizations in genomic selection.

We identified three private base-pair substitutions in exon 23 of the *CLCN7* gene, for which affected calves were homozygous. These mutations lie within a 7-bp segment. They most probably originated from a unique mutational event, possibly in the germline of one of the parents of the common ancestor to whom we could trace back all 63 affected calves. Indeed, it was recently shown (Schrider et al., 2011) that up to 3% of *de novo* mutations in the germline are part of a multinucleotide mutational event (MME).

Since mapping the gene underlying the severe pathology of the calves in 2009, we first developed an indirect, haplotype-based diagnostic test. After the identification of the mutation in 2010, we established a direct test to detect carriers among artificial insemination sires. The successive exploitation of these tests to avoid carrier mating immediately dropped the number of mutant births (supplementary material Fig. S5). The mutation-based diagnostic test has been integrated into the BBCB breeding scheme and is now systematically used to assess carrier-free status before allowing registration of new artificial insemination sires. More than 15,000 animals, mainly males, were genotyped to exclude carrier bulls from breeding and this contributed to reduce the carrier frequency from 13% in 2009 to ∼6% nowadays (supplementary material Fig. S5). Retrospective evaluation of the indirect test performance on 500 animals reanalyzed with the mutation-based test identified a single false positive (haplotype identical to the disease haplotype based on markers but without the mutation) and no false negatives (carrier of the mutation on a recombinant haplotype). It further demonstrated the practical interest of developing an indirect diagnostic test while hunting for the causative mutation. In the Belgian Blue cattle breed, diagnostic test diffusion, currently for seven recessive diseases, has directly influenced calf mortality rate (before 6 months), lowering it from 11% in 2006 to an estimated 7% in 2012. This reduction has had a huge economic impact that is widely recognized by breeders and veterinarians. However, due to the avoidance of carrier mating, we unfortunately lacked tissue from more mutant calves for further analysis. For example, we had no brain sample that could be processed for histological analysis.

### Osteopetrotic phenotype of the mutant calves

Osteopetrosis has been observed in various cattle breeds. The affected calves are generally born premature (mostly stillborn). Common phenotypes include an abnormal skull shape, inferior brachygnatia (shortened mandible) and a protruding tongue, features also observed with the calves homozygous for the *CLCN7* mutation. Recently, a deletion mutation in the *SLC4A2* gene, encoding the anion exchanger AE2, has been found to underlie osteopetrosis in Red Angus cattle (Meyers et al., 2010). By mediating Cl^−^/HCO_3_^−^ exchange across the basolateral membrane, AE2 leads to acid uptake into the cytosol (Coury et al., 2013; Wu et al., 2008), which is then extruded by the H^+^-ATPase of the ruffled border into the resorption lacuna. ClC-7 is thought to enable proton pumping by providing an electric shunt for the proton pump (Kornak et al., 2001). In addition, ClC-7 might be required for the exocytic insertion of proton pumps and other constituents of lysosomal membranes into the ruffled border (Stauber and Jentsch, 2013), a notion supported by the underdevelopment of the ruffled border observed in *Clcn7*^−/−^ mice (Kornak et al., 2001). The hepatomegaly found in most affected calves can be explained by an extramedullary blood production secondary to the osteopetrotic obliteration of the bone marrow cavity (Tolar et al., 2004), and – as mentioned above – the hamartomas could result from the lack of tooth eruption accompanying the osteopetrosis.

### A physiological function of slow gating?

Like several human osteopetrosis-associated *CLCN7* mutations (Leisle et al., 2011), the present bovine *CLCN7* mutation drastically accelerated ClC-7/Ostm1 gating. The acceleration by a factor of about three is comparable with that of other mutations that accelerate ClC-7/Ostm1 gating by a factor between ~2.5 and >5 (Leisle et al., 2011; Ludwig et al., 2013) and is clearly stronger than the less-than-twofold acceleration by a recently identified human ClC-7 mutation causative for osteopetrosis (Barvencik et al., 2013). Most of those mutations affected residues that might interfere with contacts of cytosolic CBS domains and the ClC-7 transmembrane block (Leisle et al., 2011). We have recently shown that the slow gating kinetics of ClC-7/Ostm1 is based on the common gating of both dimer subunits, which depends on the cytosolic C-terminus (Ludwig et al., 2013). The mutation identified in this study localizes in CBS2 close to the interface with the CBS2 domain of the other ClC-7 subunit and might impinge on the common gating mechanism. None of these mutations significantly altered the voltage-dependence of the currents or the subcellular localization in heterologous expression systems (Barvencik et al., 2013; Leisle et al., 2011). These observations suggest that the unique slow kinetics of voltage-activation of ClC-7/Ostm1 might be crucial for its cellular function. However, before drawing this conclusion it must be ascertained that these mutations do not change the abundance, localization or other properties of ClC-7 *in vivo*.

Acceleration of ClC-7 gating kinetics has been found with mutations identified both in autosomal recessive osteopetrosis (ARO) and in autosomal dominant osteopetrosis type 2 (ADO2) (Leisle et al., 2011). ADO2 patients present a mild osteopetrosis that becomes apparent in adulthood. Because heterozygous *Clcn7*^+/−^ mice suggest that osteopetrosis cannot be caused by *CLCN7* haplo-insufficiency, these mutations are likely to exert a dominant effect. Dominance could result for example by ER retention of a wild-type/mutant heterodimer, which could reduce ClC-7 activity down to 25%. It might also be explained by a weak gain-of-function effect (more current after short times of depolarization due to accelerated gating) that would be fully expressed in mutant/mutant homodimers that are expected to account for 25% of total ClC-7 dimers in heterozygous patients, but would be less pronounced in the expected 50% wild-type/mutant heteromers because the presence of an attached wild-type subunit reduces the gating kinetics of the fast mutants (Ludwig et al., 2013). Obviously, for either mechanism (ER retention or gain of current) the same mutation will have more severe effects when present on both alleles.

Although the acceleration of ClC-7/Ostm1 that we observed previously with several pathogenic human mutations (Leisle et al., 2011) is intriguing, we could not exclude the possibility that these mutations caused osteopetrosis by reducing ClC-7 protein levels *in vivo*. So far, we had protein data only for one such accelerating mutant (human ClC-7^R762Q^) (Leisle et al., 2011), which showed that the mutant protein is unstable in patient-derived fibroblasts (Kornak et al., 2001). Here we show for the first time that a ‘fast’ osteopetrosis-causing ClC-7 mutant displays near-normal expression levels and lysosomal localization in native tissues, strengthening the case for a pathogenic role of accelerated gating. However, it remains unclear how such ‘gain-of-function’ mutations might cause very similar, if not identical phenotypes as a loss-of-function, as observed with *Clcn7*^−/−^ mice and several human mutations.

The related endosomal Cl^−^/H^+^ exchangers ClC-3 through ClC-6 display much faster gating kinetics, with a large current component being instantaneously present upon depolarization (Friedrich et al., 1999; Li et al., 2000; Neagoe et al., 2010). It remains to be clarified whether the voltage dependence of intracellular CLCs is the same on their native compartments as at the plasma membrane, where they can be analyzed. An outwardly rectifying ClC-7/Ostm1 should be largely inactive at the reported inside-positive voltage of lysosomes (Koivusalo et al., 2011; Leisle et al., 2011). Reductionist model calculations of vesicular acidification, however, predict an inside-negative voltage generated by pH gradient-driven Cl^−^ import (Weinert et al., 2010). The importance of slow ClC-7/Ostm1 gating suggests that the exchanger should be inert to relatively quick voltage transitions, which could be envisaged upon triggered release of luminal calcium or during fusion processes with endosomal compartments. To further approach this interesting issue, new tools to monitor lysosomal voltage and ClC-7/Ostm1 activity will be required. Because ion transport by ClC-7/Ostm1 will affect lysosomal ion homeostasis in general and the voltage and osmolarity of this compartment in particular (Stauber and Jentsch, 2013), it remains to be elucidated how an acceleration of the ClC-7/Ostm1 gating impinges upon these parameters.

## MATERIALS AND METHODS

### Genome-wide haplotype-based association study

DNA extraction and SNP genotyping using a custom-made bovine 50K SNP array were conducted using standard procedures as described (Charlier et al., 2008). Haplotypes were reconstructed using Beagle (Browning and Browning, 2007). DualPHASE was then used to assign haplotypes to ten hidden haplotype states (ancestral haplotypes) (Druet and Georges, 2010). Genome-wide association mapping was performed using GLASCOW (Zhang et al., 2012): association between the hidden haplotype states and the phenotype was tested using a generalized linear mixed model (with a logit link function) including a polygenic effect accounting for stratification. The genomic relatedness among individuals was estimated on the basis of hidden haplotype state similarity.

### Genome-wide resequencing

Four affected individuals homozygous for the defined IBD haplotype were selected, as well as eight unrelated individuals from the same breed, genotyped as non-carriers of the disease haplotype. To minimize the sequencing cost and maximize the putative applied outcome, the eight control individuals were chosen as being homozygous mutant for two additional distinct recessive diseases (four mutants each) for which the chromosomal location was known but the causative genes and mutations were still to be found. The Pair-End Library Prep Kit v2 from Illumina was used to generate a 200–1250 bp paired-end sequencing library from genomic DNA fragments for each animal. Briefly, total genomic DNA was extracted and fragmented by sonication (Bioruptor, Diagenode). Size-selected fragments were end-repaired and ligated to Illumina Paired-End sequencing adapters. Each library was sequenced on one lane of the flow-cell of an Illumina GAIIx with the paired-end module to generate high-quality reads (2×110 bp). Reads were mapped and analyzed with publicly available software: Burrows-Wheeler Alignment Tool (http://bio-bwa.sourceforge.net) and SAMtools (http://samtools.sourceforge.net). The output files were readily uploaded in the Integrative Genomics Viewer (IGV) (Robinson et al., 2011) and visually scrutinized for private variation.

### Mutation validation at the DNA and mRNA levels

A primer pair (gUP1-gDN1) was designed to amplify a PCR product encompassing the three private SNP identified by NGS (supplementary material Table S4). It was used to amplify products from genomic DNA of homozygous cases, carriers and unaffected unrelated individuals using standard procedures. Amplicons were directly sequenced using the Big Dye terminator cycle sequencing kit (Applied Biosystems, Foster City, CA). Electrophoresis of purified sequencing reactions was performed on an ABI PRISM 3730 DNA analyzer (PE Applied Biosystems, Foster City, CA). Sequence traces were aligned and compared with bovine reference using the Phred/Phrap/Consed package (www.phrap.org/phredphrapconsed.html). Total RNA was extracted from kidney of one homozygous case and one unaffected unrelated individual using Trizol (Invitrogen). The obtained RNA was treated with TurboDNaseI (Ambion) and cDNA was synthesized using Superscript™III First Strand Synthesis System for RT-PCR (Invitrogen). Full length *CLCN7* cDNA was amplified using three specific primer pairs (cUP1-cDN1, cUP2-cDN2, cUP3-cDN3) (supplementary material Table S4) and the three overlapping amplicons were directly sequenced and analyzed as describe above.

### Development of a genotyping test for the missense mutation

A 5′ exonuclease assay (Taqman) was developed to genotype the *[c2248T >C + c2250C >A] CLCN7* mutations, using 5′-CCATGGACCTGT -CTGAGTTCAT-3′ and 5′-ACCCCCCAGCAGTACCT-3′ as PCR primer pair combined with 5′-CCC[TAC]ACGGTGCCC-3′ (wild type) and 5′-CC[CAA]ACGGTGCCC-3′ (mutant), respectively, labeled with VIC and FAM as Taqman probes. Allelic discrimination reactions were carried out on an ABI7900HT instrument (Applied Biosystems, Foster City, CA) using standard procedures.

### Western blotting

Tissues from calves and mice (kidney or cerebellum) were homogenized in HEPES-buffered saline (HBS, pH 7.4) with protease inhibitors (complete protease inhibitor cocktail, Roche) and cleared by two successive centrifugation steps at 1000 ***g*** for 10 minutes. Membrane protein fractions were prepared by centrifugation at 100,000 ***g*** for 30 minutes and subsequent resuspension in HBS supplemented with 2% (w/v) SDS. Membrane protein fraction (80 μg per sample) or whole-organ lysate (60 μg per sample) were separated by SDS-PAGE and transferred to nitrocellulose. ClC-7 was detected with a polyclonal rabbit antibody directed against the C-terminal peptide RFPPIQSIHVSQDEREC (100% conserved between murine and bovine ClC-7 protein). The other primary antibodies were guinea-pig anti-Ostm1 (Lange et al., 2006), rabbit antibodies against subunit c of the mitochondrial ATP synthase (a gift from Eiki Kominami, Juntendo University, Tokyo, Japan), cathepsin D (Calbiochem) and LC3B (Cell Signaling), and a mouse antibody against α-tubulin (clone DM1A, Sigma). After incubation with secondary antibodies conjugated to horseradish peroxidase (Jackson ImmunoResearch), chemiluminescence signal was detected with a camera system (PeqLab).

### Expression constructs

Constructs for expression of rat ClC-7 (Kornak et al., 2001) and of fluorescently tagged Ostm1-GFP (Stauber and Jentsch, 2010) in cell culture were as described. Constructs in pTLN for the expression of partially cell surface-localized human ClC-7^L23A,L24A,L68A,L69A^ (hClC-7^PM^) and Ostm1 in *Xenopus* oocytes, were described previously (Leisle et al., 2011). Point mutations were introduced by PCR and the complete ORF of all constructs were confirmed by sequencing.

### Subcellular targeting and interaction with Ostm1

HeLa cells were transfected with plasmid DNA encoding the respective construct(s) using FuGENE6 (Roche). ClC-7 with Ostm1-GFP constructs were co-transfected at a 50:1 (ClC-7:Ostm1-GFP) weight ratio to prevent expression of excess Ostm1-GFP, which would not be exported from the ER. Cells were grown for 26–128 hours before fixation with 4% PFA in PBS for 15 minutes. After incubation with 30 mM glycine in PBS for 5 minutes, cells were incubated sequentially with primary and secondary antibodies in PBS containing 0.1% saponin supplemented with 5% BSA. Each incubation was for 1 hour at room temperature. Primary antibodies were rabbit anti-ClC-7 (7N4B) (Kornak et al., 2001), and mouse anti-Lamp-1 (clone H4A3; from the DSHB). Secondary antibodies conjugated to AlexaFluor 488, 546 or 633 were from Molecular Probes. Images were acquired with an LSM510 confocal microscope with a 63×, 1.4 NA oil-immersion lens (Zeiss).

### Two-electrode voltage clamp measurements

Current measurements of ClC-7^PM^/Ostm1 were performed as described (Leisle et al., 2011). *Xenopus laevis* oocytes were injected with cRNA encoding for the hClC-7^PM^ construct and for Ostm1 (23 ng each), which was transcribed with the mMessage Machine Kit (Ambion) after linearizing of the pTLN plasmid with *Mlu*I. After 3 days incubation in ND96 saline (96 mM NaCl, 2 mM K-gluconate, 1.8 mM Ca-gluconate, 1 mM Mg-gluconate and 5 mM HEPES, pH 7.5) at 17°C, currents were measured in ND96 using a standard two-electrode voltage clamp at room temperature, employing a TurboTEC amplifier (npi electronic) and pClamp10.2 software (Molecular Devices). The holding potential was −30 mV. Test pulses of 2 seconds between −80 and +80 mV (in 20-mV steps) were followed by a 0.5-second deactivation pulse at −80 mV. Rate constants of the activation kinetics were determined by fitting the currents of the first 250 milliseconds of depolarization to +80 mV to a single-exponential function.

## Supplementary Material

Supplementary Material
