# Supplementary material for: A missense mutation accelerating the gating of the lysosomal Cl−/H+-exchanger ClC-7/Ostm1 causes osteopetrosis with gingival hamartomas in cattle
Source: Dis Model Mech. 2013 Oct 23;7(1):119–28. doi: 10.1242/dmm.012500 (PMC3882054; doi:10.1242/dmm.012500)
Supplement: Supplementary Material [file supp_7_1_119__index.html]

A missense mutation accelerating the gating of the lysosomal Cl−/H+-exchanger ClC-7/Ostm1 causes osteopetrosis with gingival hamartomas in cattle — Supplementary Material 

# A missense mutation accelerating the gating of the lysosomal Cl−/H+-exchanger ClC-7/Ostm1 causes osteopetrosis with gingival hamartomas in cattle

## DMM012500 Supplementary Material

**Files in this Data Supplement:**

- **Supplementary Material PDF**
