# Supplementary material for: A missense mutation accelerating the gating of the lysosomal Cl−/H+-exchanger ClC-7/Ostm1 causes osteopetrosis with gingival hamartomas in cattle
Source: Dis Model Mech. 2013 Oct 23;7(1):119–28. doi: 10.1242/dmm.012500 (PMC3882054; doi:10.1242/dmm.012500)

SUPPLEMENTARY MATERIAL

Table S1. Summary of clinical symptoms. The clinical symptoms for the 63 cases are depicted in this table. '–' indicates that no data are available.

Case number	Gender	Days before term	Size at birth	Alive/dead at birth	Abnormal skull shape	Inferior brachygnatia	Hamartome (diameter)	Protruding tongue	Blindness	Abdominal hydrops	Liver hypertrophy	Kidney hypertrophy	Hydramnios	Osteopetrosis
1	F	12	small	dead	yes	yes	3 cm	yes	-	yes	yes	yes	yes	-
2	F	0	normal	dead	yes	yes	4 cm	-	-	yes	yes	yes	yes	-
3	M	0	normal	dead	yes	yes	10 cm	-	-	yes	yes	no	yes	-
4	F	15	small	dead	yes	yes	10 cm	-	-	yes	yes	yes	yes	-
5	M	30	small	dead	yes	yes	7 cm	-	-	yes	yes	yes	yes	-
6	F	0	small	alive	yes	yes	5 cm	no	yes	no	no	no	no	-
7	F	0	normal	alive	yes	yes	2 cm	no	yes	no	no	no	no	-
8	F	0	normal	alive	yes	yes	3 cm	yes	yes	no	no	no	no	-
9	F	-	-	dead	yes	yes	yes	-	-	-	-	-	-	-
10	-	0	small	dead	yes	yes	5 cm	-	-	yes	yes	yes	yes	-
11	F	0	small	dead	yes	yes	12 cm	-	-	yes	yes	yes	yes	-
12	F	0	small	alive	yes	yes	no	yes	yes	no	no	no	no	-
13	M	0	normal	alive	yes	yes	no	yes	yes	-	-	-	-	-
14	M	31	small	dead	yes	yes	10 cm	-	-	yes	yes	yes	yes	-
15	F	0	normal	alive	yes	yes	2 cm	yes	yes	no	-	-	no	-
16	F	15	small	dead	yes	yes	10 cm	-	-	yes	yes	yes	yes	-
17	M	0	normal	alive	yes	yes	12 cm	yes	yes	no	no	no	yes	-
18	F	16	small	dead	yes	yes	6 cm	-	-	yes	yes	yes	yes	-
19	M	0	small	alive	yes	yes	no	yes	yes	no	-	-	no	-
20	M	-	small	dead	yes	yes	7 cm	-	-	yes	yes	yes	yes	-
21	M	-	small	dead	yes	yes	2 cm	-	-	yes	yes	yes	yes	-
22	F	-	small	dead	yes	yes	10 cm	-	-	yes	yes	yes	yes	-
23	F	-	-	dead	yes	yes	yes	-	-	yes	-	-	yes	-
24	M	0	normal	dead	yes	yes	3 cm	-	-	yes	yes	no	no	-
25	M	60	small	dead	yes	yes	5 cm	-	-	yes	yes	yes	yes	-
26	M	0	normal	dead	yes	yes	yes	-	-	-	-	-	yes	-
27	M	0	normal	dead	yes	yes	yes	-	-	-	-	-	-	-
28	F	-	-	dead	yes	yes	yes	-	-	-	-	-	-	-
29	M	30	small	dead	yes	yes	yes	-	-	yes	-	-	yes	-
30	F	21	small	dead	yes	yes	4 cm	-	-	yes	yes	yes	yes	-
31	M	0	normal	alive	yes	yes	3 cm	-	yes	no	-	-	no	-
32	M	-	-	dead	yes	yes	yes	-	-	-	-	-	-	-
33	F	7	small	dead	yes	yes	10 cm	-	-	yes	yes	yes	yes	-
34	M	0	normal	dead	yes	yes	yes	-	-	yes	-	-	yes	-

35	M	-	-	dead	yes	yes	yes	-	-	-	-	-	-	-
36	M	0	normal	alive	yes	yes	no	-	yes	no	-	-	no	-
37	M	15	small	dead	yes	yes	1 cm	-	-	yes	-	-	yes	-
38	-	0	normal	alive	yes	yes	no	no	yes	no	-	-	no	-
39	-	0	normal	alive	yes	yes	no	yes	yes	no	-	-	no	-
40	-	-	-	dead	yes	yes	yes	-	-	-	-	-	-	-
41	F	30	small	dead	yes	yes	4 cm	-	-	yes	yes	yes	yes	-
42	-	-	-	dead	yes	yes	yes	-	-	-	-	-	-	-
43	-	0	normal	dead	yes	yes	yes	-	-	no	-	-	no	-
44	-	0	small	dead	yes	yes	10 cm	-	-	yes	yes	no	yes	-
45	M	0	normal	alive	yes	yes	no	-	yes	no	-	-	no	-
46	-	-	-	dead	yes	yes	yes	-	-	-	-	-	-	-
47	-	0	normal	alive	yes	yes	no	yes	yes	no	-	-	no	-
48	-	-	-	dead	yes	yes	yes	-	-	-	-	-	-	-
49	F	0	small	dead	yes	yes	10 cm	-	-	no	-	-	no	-
50	M	0	normal	dead	yes	yes	2 cm	-	-	no	-	-	no	-
51	M	30	small	dead	yes	yes	5 cm	-	-	yes	-	-	yes	-
52	-	-	-	dead	yes	yes	yes	-	-	-	-	-	-	-
53	-	-	-	dead	yes	yes	yes	-	-	-	-	-	-	-
54	M	0	normal	alive	yes	yes	no	yes	yes	no	-	-	no	-
55	M	0	normal	dead	yes	yes	yes	-	-	yes	-	-	yes	-
56	F	0	normal	dead	yes	yes	yes	-	-	yes	-	-	yes	-
57	F	0	normal	dead	yes	yes	yes	-	-	yes	-	-	yes	-
58	M	0	normal	alive	yes	yes	yes	-	yes	no	-	-	no	-
59	M	0	normal	dead	yes	yes	yes	-	-	yes	-	-	yes	-
60	F	0	normal	alive	yes	yes	yes	yes	yes	no	yes	no	no	yes
61	M	0	small	dead	yes	yes	no	-	-	-	yes	-	-	yes
62	M	0	normal	alive	yes	yes	no	yes	yes	no	yes	no	no	yes
63	F	0	normal	dead	yes	yes	no	yes	-	yes	yes	no	yes	yes

Figure S1. Gene content of the non-recombinant autozygous interval. Screen capture of the Ensembl Genome Browser (http://www.ensembl.org/Bos_taurus/Info/Index) displaying the 82 annotated bovine transcripts in the corresponding 1.15 Mb region.

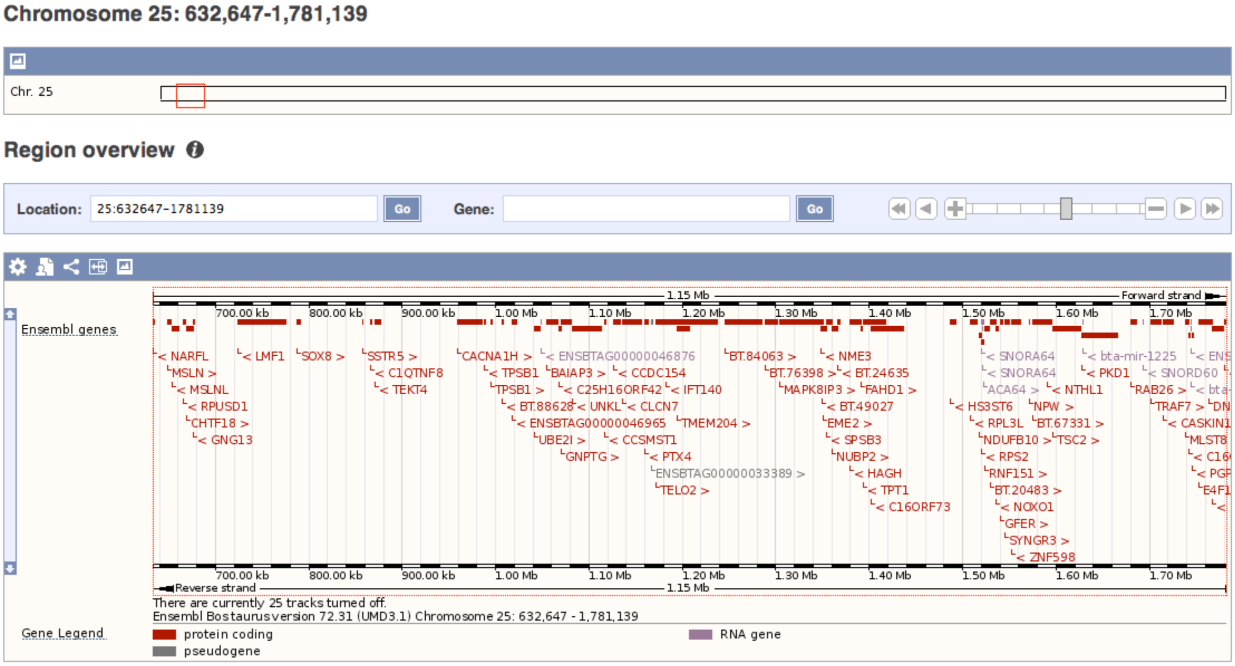

Table S2. List of annotated transcripts the 1.15 Mb non-recombinant autozygous interval on BTA25. The list of 82 transcripts was downloaded from the Ensembl Genome Browser web site (http://www.ensembl.org/Bos_taurus/Info/Index). Gene coordinates are given for bovine genome assembly UMD3.1. The *CLCN7* gene is highlighted in red.

Start (bp)	End (bp)	Str	Name	Transcript ID	Description
627456	633977	-1	*NARFL*	ENSBTAT00000003225	Cytosolic Fe-S cluster assembly factor NARFL [Source:UniProtKB/Swiss-Prot;Acc:A4FV58]
649253	652835	1	*MSLN*	ENSBTAT00000000202	mesothelin precursor [Source:RefSeq peptide;Acc:NP_001093844]
653105	660655	-1	*MSLNL*	ENSBTAT00000047719	Uncharacterized protein [Source:UniProtKB/TrEMBL;Acc:F1MQ52]
665058	668257	-1	*RPUSD1*	ENSBTAT00000000204	RNA pseudouridylate synthase domain-containing protein 1 [Source:UniProtKB/Swiss-Prot;Acc:Q17QT4]
668529	676616	1	*CHTF18*	ENSBTAT00000026311	chromosome transmission fidelity protein 18 homolog [Source:RefSeq peptide;Acc:NP_001179389]
676665	677047	-1	*GNG13*	ENSBTAT00000026313	guanine nucleotide-binding protein G(I)/G(S)/G(O) subunit gamma-13 [Source:RefSeq peptide;Acc:NP_001193261]
724446	775899	-1	*LMF1*	ENSBTAT00000004206	Lipase maturation factor 1 [Source:UniProtKB/Swiss-Prot;Acc:Q0P5C0]
787493	790981	1	*SOX8*	ENSBTAT00000027633	Uncharacterized protein [Source:UniProtKB/TrEMBL;Acc:F1MBL5]
857167	858273	1	*SSTR5*	ENSBTAT00000052225	Somatostatin receptor type 5 [Source:UniProtKB/Swiss-Prot;Acc:F1MV99]
865752	867111	-1	*C1QTNF8*	ENSBTAT00000016731	Uncharacterized protein [Source:UniProtKB/TrEMBL;Acc:E1BF11]
871131	877122	-1	*TEKT4*	ENSBTAT00000016726	Tektin-4 [Source:UniProtKB/Swiss-Prot;Acc:Q2TA38]
959758	984950	1	*CACNA1H*	ENSBTAT00000012991	Uncharacterized protein [Source:UniProtKB/TrEMBL;Acc:F1MQV2]
987390	989247	-1	*TPSB1*	ENSBTAT00000009636	TPSB1 protein; Uncharacterized protein [Source:UniProtKB/TrEMBL;Acc:A6QPI9]
994572	996276	1	*TPSB1*	ENSBTAT00000037579	tryptase beta-2 precursor [Source:RefSeq peptide;Acc:NP_776627]
1006912	1008669	-1	*BT.88628*	ENSBTAT00000066317	Uncharacterized protein [Source:UniProtKB/TrEMBL;Acc:G3MXR7]
1017616	1022334	-1		ENSBTAT00000063348	Uncharacterized protein [Source:UniProtKB/TrEMBL;Acc:G3MYJ4]
1041085	1048106	1	*UBE2I*	ENSBTAT00000056739	SUMO-conjugating enzyme UBC9 [Source:RefSeq peptide;Acc:NP_001092842]
1048611	1048737	-1		ENSBTAT00000063629	
1054501	1066815	1	*BAIAP3*	ENSBTAT00000018748	Uncharacterized protein [Source:UniProtKB/TrEMBL;Acc:F1MNU6]
1067758	1070205	-1	*C25H16ORF42*	ENSBTAT00000018749	probable ribosome biogenesis protein C16orf42 homolog [Source:RefSeq peptide;Acc:NP_001092381]
1070287	1081303	1	*GNPTG*	ENSBTAT00000018766	N-acetylglucosamine-1-phosphotransferase subunit gamma [Source:UniProtKB/Swiss-Prot;Acc:Q58CS8]
1082676	1113094	-1	*UNKL*	ENSBTAT00000018773	Uncharacterized protein [Source:UniProtKB/TrEMBL;Acc:F1MNI6]
1116677	1117637	-1	*CCSMST1*	ENSBTAT00000047510	Protein CCSMST1 [Source:UniProtKB/Swiss-Prot;Acc:Q1ECT8]
1126769	1133966	-1	*CCDC154*	ENSBTAT00000000741	Uncharacterized protein [Source:UniProtKB/TrEMBL;Acc:F1MS01]

**1135571**	**1156102**	**-1**	***CLCN7***	**ENSBTAT00000021122**	**H(+)/Cl(-) exchange transporter 7 [Source:UniProtKB/Swiss-Prot;Acc:Q4PKH3]**
1159757	1163098	-1	*PTX4*	ENSBTAT00000006786	Uncharacterized protein [Source:UniProtKB/TrEMBL;Acc:E1B8G2]
1167351	1168312	1		ENSBTAT00000049975	
1172266	1181534	1	*TELO2*	ENSBTAT00000025412	Uncharacterized protein [Source:UniProtKB/TrEMBL;Acc:E1B7I8]
1182105	1237269	-1	*IFT140*	ENSBTAT00000047406	Uncharacterized protein [Source:UniProtKB/TrEMBL;Acc:E1B860]
1194760	1207350	1	*TMEM204*	ENSBTAT00000009534	Transmembrane protein 204 [Source:UniProtKB/Swiss-Prot;Acc:Q0IIE5]
1245669	1284542	1	*BT.84063*	ENSBTAT00000002854	protein cramped-like [Source:RefSeq peptide;Acc:NP_001192803]
1288766	1301379	1	*BT.76398*	ENSBTAT00000064148	hematological and neurological expressed 1-like protein [Source:RefSeq peptide;Acc:NP_001075015]
1288766	1301379	1	*BT.76398*	ENSBTAT00000002860	hematological and neurological expressed 1-like protein [Source:RefSeq peptide;Acc:NP_001075015]
1304435	1348409	1	*MAPK8IP3*	ENSBTAT00000002865	C-Jun-amino-terminal kinase-interacting protein 3 [Source:RefSeq peptide;Acc:NP_001069564]
1348415	1349600	-1	*NME3*	ENSBTAT00000022012	nucleoside diphosphate kinase 3 precursor [Source:RefSeq peptide;Acc:NP_001092456]
1349893	1351145	-1	*BT.49027*	ENSBTAT00000037541	28S ribosomal protein S34, mitochondrial [Source:RefSeq peptide;Acc:NP_001030577]
1351233	1354061	1	*EME2*	ENSBTAT00000022021	Uncharacterized protein [Source:UniProtKB/TrEMBL;Acc:E1B7C9]
1351233	1354061	1	*EME2*	ENSBTAT00000063538	Uncharacterized protein [Source:UniProtKB/TrEMBL;Acc:E1B7C9]
1351233	1354061	1	*EME2*	ENSBTAT00000066331	Uncharacterized protein [Source:UniProtKB/TrEMBL;Acc:E1B7C9]
1354553	1360122	-1	*SPSB3*	ENSBTAT00000022024	SPRY domain-containing SOCS box protein 3 [Source:UniProtKB/Swiss-Prot;Acc:Q3MHZ2]
1360579	1365802	1	*NUBP2*	ENSBTAT00000022029	Cytosolic Fe-S cluster assembly factor NUBP2 [Source:UniProtKB/Swiss-Prot;Acc:Q3MHY6]
1366647	1368479	-1	*BT.24635*	ENSBTAT00000047326	insulin-like growth factor-binding protein complex acid labile subunit precursor [Source:RefSeq peptide;Acc:NP_001069431]
1379295	1391295	-1	*HAGH*	ENSBTAT00000026635	Hydroxyacylglutathione hydrolase, mitochondrial [Source:UniProtKB/Swiss-Prot;Acc:Q3B7M2]
1391462	1393086	1	*FAHD1*	ENSBTAT00000046873	Acylpyruvase FAHD1, mitochondrial [Source:UniProtKB/Swiss-Prot;Acc:Q2HJ98]
1394201	1417716	-1	*TPT1*	ENSBTAT00000065799	Translationally-controlled tumor protein [Source:UniProtKB/Swiss-Prot;Acc:Q5E984]
1402494	1436118	-1	*C16ORF73*	ENSBTAT00000037536	Uncharacterized protein [Source:UniProtKB/TrEMBL;Acc:E1BA02]
1402494	1436118	-1	*C16ORF73*	ENSBTAT00000065143	Uncharacterized protein [Source:UniProtKB/TrEMBL;Acc:E1BA02]
1486479	1492437	-1	*HS3ST6*	ENSBTAT00000025630	Uncharacterized protein [Source:UniProtKB/TrEMBL;Acc:E1B729]
1508568	1515379	-1	*RPL3L*	ENSBTAT00000012541	60S ribosomal protein L3-like [Source:UniProtKB/Swiss-Prot;Acc:Q3SZ10]
1517626	1520287	1	*NDUFB10*	ENSBTAT00000012542	NADH dehydrogenase [ubiquinone] 1 beta subcomplex subunit 10 [Source:UniProtKB/Swiss-Prot;Acc:Q02373]
1520493	1522670	-1	*RPS2*	ENSBTAT00000012544	40S ribosomal protein S2 [Source:UniProtKB/Swiss-Prot;Acc:O18789]
1520844	1520974	-1	*SNORA64*	ENSBTAT00000059386	Small nucleolar RNA SNORA64/SNORA10 family [Source:RFAM;Acc:RF00264]
1521490	1521623	-1	*SNORA64*	ENSBTAT00000059680	Small nucleolar RNA SNORA64/SNORA10 family [Source:RFAM;Acc:RF00264]

1522971	1523097	1	*ACA64*	ENSBTAT00000062760	Small nucleolar RNA ACA64 [Source:RFAM;Acc:RF01225]
1524318	1528358	1	*RNF151*	ENSBTAT00000047302	RING finger protein 151 [Source:UniProtKB/Swiss-Prot;Acc:Q2TBT8]
1529747	1535805	1	*BT.20483*	ENSBTAT00000027147	transducin beta-like protein 3 [Source:RefSeq peptide;Acc:NP_001040084]
1536097	1538058	-1	*NOXO1*	ENSBTAT00000047285	Uncharacterized protein [Source:UniProtKB/TrEMBL;Acc:E1BAA4]
1540863	1543188	1	*GFER*	ENSBTAT00000023446	FAD-linked sulfhydryl oxidase ALR [Source:RefSeq peptide;Acc:NP_001180117]
1545800	1549999	1	*SYNGR3*	ENSBTAT00000011211	Synaptogyrin-3 [Source:UniProtKB/Swiss-Prot;Acc:A2VE58]
1552258	1562560	-1	*ZNF598*	ENSBTAT00000001356	Uncharacterized protein [Source:UniProtKB/TrEMBL;Acc:E1B928]
1571169	1571725	1	*NPW*	ENSBTAT00000064568	Uncharacterized protein [Source:UniProtKB/TrEMBL;Acc:G3MWV7]
1575649	1589619	1	*BT.67331*	ENSBTAT00000065244	Na(+)/H(+) exchange regulatory cofactor NHE-RF2 [Source:RefSeq peptide;Acc:NP_001070533]
1575649	1589619	1	*BT.67331*	ENSBTAT00000049790	Na(+)/H(+) exchange regulatory cofactor NHE-RF2 [Source:RefSeq peptide;Acc:NP_001070533]
1590252	1595934	-1	*NTHL1*	ENSBTAT00000049780	Endonuclease III-like protein 1 [Source:UniProtKB/Swiss-Prot;Acc:Q2KID2]
1596730	1626967	1	*TSC2*	ENSBTAT00000049485	Uncharacterized protein [Source:UniProtKB/TrEMBL;Acc:E1BNT2]
1627978	1666088	-1	*PKD1*	ENSBTAT00000027480	Uncharacterized protein [Source:UniProtKB/TrEMBL;Acc:E1BC86]
1628447	1628539	-1	*bta-mir-1225*	ENSBTAT00000054195	bta-mir-1225 [Source:miRBase;Acc:MI0010452]
1680110	1686192	1	*RAB26*	ENSBTAT00000000348	Ras-related protein Rab-26 [Source:UniProtKB/Swiss-Prot;Acc:Q29RR0]
1693737	1693819	-1	*SNORD60*	ENSBTAT00000059743	Small nucleolar RNA SNORD60 [Source:RFAM;Acc:RF00271]
1702116	1712907	1	*TRAF7*	ENSBTAT00000025393	E3 ubiquitin-protein ligase TRAF7 [Source:RefSeq peptide;Acc:NP_001019692]
1714641	1725252	-1	*CASKIN1*	ENSBTAT00000053168	Uncharacterized protein [Source:UniProtKB/TrEMBL;Acc:F1MB71]
1738258	1742107	1	*MLST8*	ENSBTAT00000053181	Target of rapamycin complex subunit LST8 [Source:UniProtKB/Swiss-Prot;Acc:Q17QU5]
1738258	1742107	1	*MLST8*	ENSBTAT00000013104	Target of rapamycin complex subunit LST8 [Source:UniProtKB/Swiss-Prot;Acc:Q17QU5]
1742073	1743503	-1	*C16ORF79*	ENSBTAT00000066147	Uncharacterized protein [Source:UniProtKB/TrEMBL;Acc:E1BH52]
1742073	1743503	-1	*C16ORF79*	ENSBTAT00000013120	Uncharacterized protein [Source:UniProtKB/TrEMBL;Acc:E1BH52]
1744661	1744720	-1		ENSBTAT00000054214	
1744908	1744984	-1	*bta-mir-2382*	ENSBTAT00000062039	bta-mir-2382 [Source:miRBase;Acc:MI0011419]
1746200	1747344	-1	*PGP*	ENSBTAT00000013127	Phosphoglycolate phosphatase [Source:UniProtKB/Swiss-Prot;Acc:Q2T9S4]
1753636	1762994	1	*E4F1*	ENSBTAT00000013136	transcription factor E4F1 [Source:RefSeq peptide;Acc:NP_001192754]
1763777	1766831	1	*DNASE1L2*	ENSBTAT00000013142	deoxyribonuclease-1-like 2 precursor [Source:RefSeq peptide;Acc:NP_001098489]

Figure S2. Identification of three private nucleotide substitutions in *CLCN7* exon 23 from whole-genome sequencing of cases. (A) Screen capture of an IGV output displaying the sequence reads from cases and controls. (B) Schematic representation of the bovine *CLCN7* genomic organization (adapted from UCSC browser) highlighting exon 23.

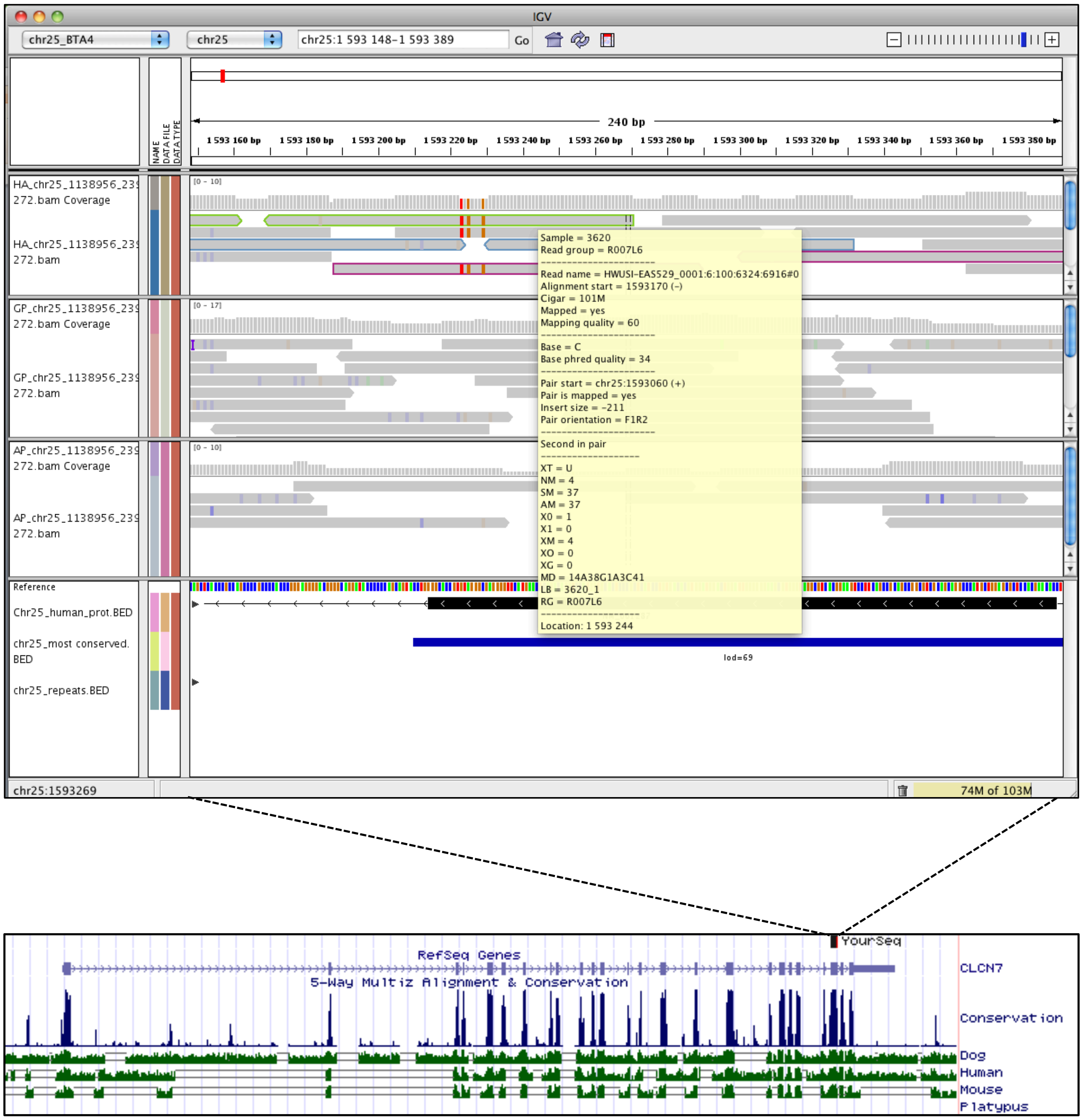

Figure S3. Position-Specific Scoring Matrix (PSSM) output for the CBS2 domain of the mutant bovine ClC-7. Alignment stack of the cd04591 domain (containing two tandem repeats of the cystathionine beta-synthase (CBS pair) domains in the EriC CLC-type chloride channels in eukaryotes and bacteria) for a 20 amino acid sequence around the Y750Q mutation in CBS2; a highly negative score (dark red) is obtained when the mutant Q750 is substituted for the wild-type Y750 (Marchler-Bauer, A., *et al.* (2011). CDD: a Conserved Domain Database for the functional annotation of proteins. *Nucleic Acids Res* 39, D225-229).

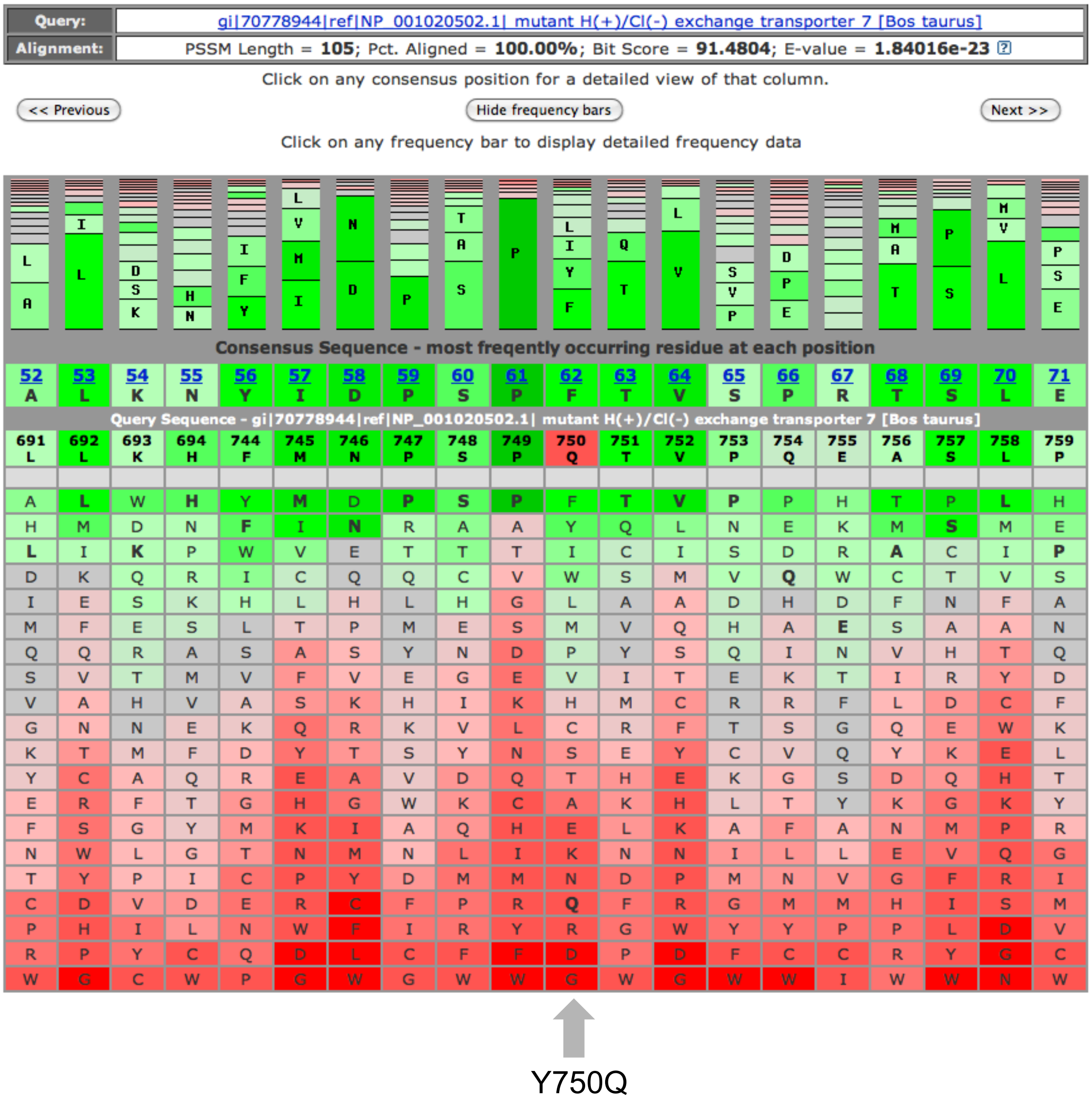

Table S3. Variations on the disease haplotype, absent from the controls. The table displays the position of each unfiltered variation within the 1.15 Mb chromosomal (Chr.) region, the respective position (Start), the allele on bovine reference sequence (Ref.), the identified derived allele (Der.), the location in respect to annotated genes (Annotation), the gene encompassing the variation (Gene) and the amino acid substitution (Effect), if any. The three substitutions originally identified in *CLCN7* are highlighted in orange.

Chr.	Start (bp)	Ref.	Der.	Annotation	Gene	Effect
25	786838	G	A	Intergenic		
25	872236	G	T	Intergenic		
25	892640	G	C	Intergenic		
25	902024	G	C	Intergenic		
25	949666	G	A	Intergenic		
25	956299	C	G	Intergenic		
25	989738	C	T	Intergenic		
25	1086081	C	G	Intronic	*UNKL*	
25	1137017	G	T	Coding	*CLCN7*	Synonymous
25	1137019	A	G	Coding	*CLCN7*	Y750Q
25	1137023	C	G	Coding	*CLCN7*	Y750Q
25	1190837	C	T	Coding	*IFT140*	R1023Q
25	1280350	C	A	Coding	*CRAMP1L*	Synonymous
25	1351987	G	A	Intronic	*EME2*	
25	1448057	G	A	Intergenic		
25	1552885	TGGAGGCTTCCGCCTTTGG	T	Intronic	*ZNF598*	

Table S4. Primer pairs for gDNA and cDNA.

Genomic DNA		
Primer name	5'-3' sequence	Size
gUP1	CATCGTCCTACTCAAGCACAAG	666 bp
gDN1	CCCCCTTTCCAAGCCGGTAC	
**cDNA**		
Primer name	5'-3' sequence	Size
cUP1	CCAACGTTTCCAAGAAGGTGTC	541 bp
cDN1	AGCAGGAGGGAGAAGGACAGTC	
cUP2	CGTGGCCTGCTTCATCGACATC	1088 bp
cDN2	AGGCCAGGAAGAAGTAGACCAG	
cUP3	TGATGGGGAGTACAACTCGATG	1256 bp
cDN3	GCAGTTGCACAGATTCCTAAAG	

Figure S4. Western blot analysis of overexpressed ClC-7/Ostm1. HeLa cells were transfected with rat ClC-7, either wild-type (WT) or mutant (MUT), and Ostm1, or with Ostm1 alone, or mock-transfected. Membrane protein-enriched lysates were prepared 26 hours after transfection and analyzed by Western blot (40 μg per lane) with antibodies against ClC-7 (7N4B, Kornak et al., Cell 2001) (A), or against Ostm1 (Lange et al., Nature 2006) (B). Immunoblotting for α-tubulin served as loading control. Overexpressed Ostm1 exists predominantly in its endoplasmic reticulum-resident, uncleaved form. Different patterning between Ostm1 alone and co-expressed may be due to different glycosylation. In the higher exposure, it is obvious that the processed, lysosomal form of Ostm1 is present when co-expressed with ClC-7, both wild-type and mutant.

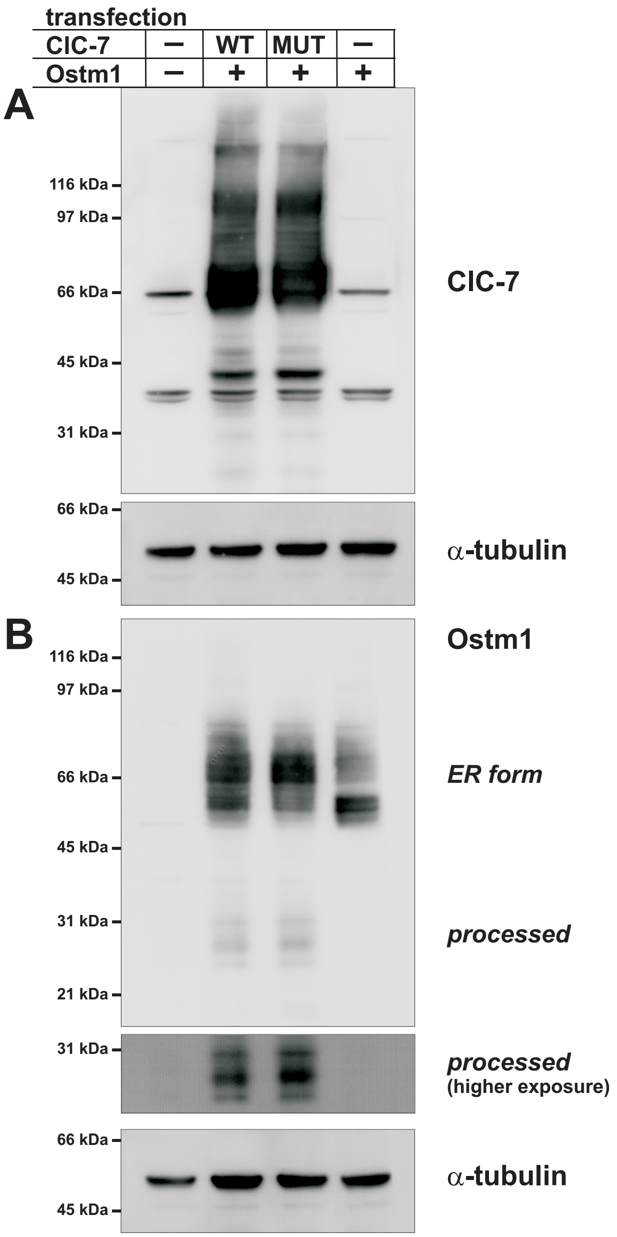

Figure S5. Yearly repartition of hamartoma cases collected and concomitant carrier frequency evolution in the healthy Belgian Blue population. Numbers of cases reported to the heredo-surveillance platform are shown in red; carrier frequency evolution, since diagnostic test availability (haplotype-based test from September 2009, replaced by a direct, mutation-based test in April 2010) until August 2013, is shown in grey; yearly number of genotyped animals is indicated as data labels.

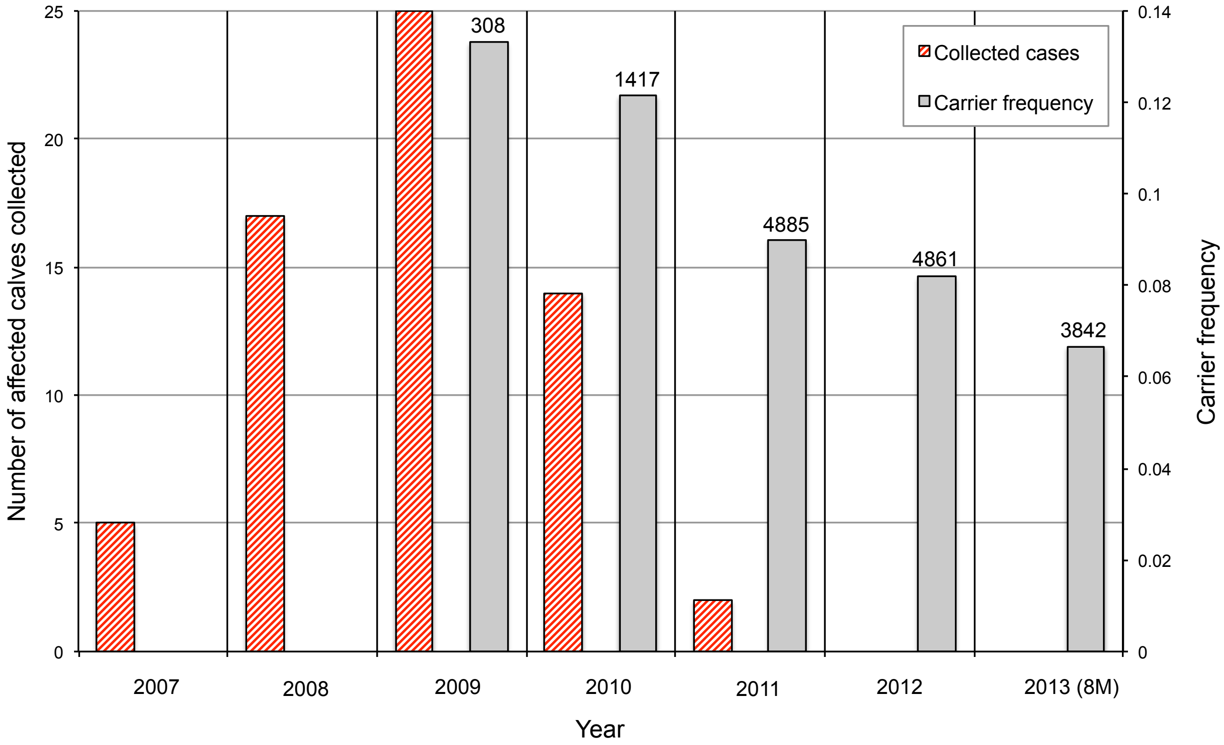

Supplement: Supplementary Material [file supp_012500_DMM012500.pdf]
